# Effects of butyrate on intestinal ischemia-reperfusion injury via the HMGB1-TLR4-MyD88 signaling pathway

**DOI:** 10.18632/aging.205797

**Published:** 2024-05-03

**Authors:** Yuanyuan Rong, Meili Xu, Tao Hu, Shasha Zhang, Jianfeng Fu, Huaqin Liu

**Affiliations:** 1 Department of Anesthesiology, The Fourth Hospital of Hebei Medical University, Shijiazhuang 050011, Hebei, China

**Keywords:** bioinformatics, butyrate, HMGB1-TLR4-MyD88 pathway, intestinal ischemia-reperfusion injury, senescence, molecular docking

## Abstract

Background: This study combined bioinformatics and experimental verification in a mouse model of intestinal ischemia-reperfusion injury (IRI) to explore the protection mechanism exerted by butyrate against IRI.

Methods: GeneCards, Bioinformatics Analysis Tool for Molecular Mechanisms of Traditional Chinese Medicine and GSE190581 were used to explore the relationship between butyrate and IRI and aging. Protein-protein interaction networks involving butyrate and IRI were constructed via the STRING database, with hub gene analysis performed through Cytoscape. Functional enrichment analysis was conducted on intersection genes. A mouse model of IRI was established, followed by direct arterial injection of butyrate. The experiment comprised five groups: normal, sham, model, vehicle, low-dose butyrate, and high-dose butyrate. Intestinal tissue observation was done via transmission electron microscopy (TEM), histological examination via hematoxylin and eosin (H&E) staining, tight junction proteins detection via immunohistochemistry, and Western blot analysis of hub genes. Drug-target interactions were evaluated through molecular docking.

Results: Butyrate protected against IRI by targeting 458 genes, including HMGB1 and TLR4. Toll-like receptor pathway was implicated. Butyrate improved intestinal IRI by reducing mucosal damage, increasing tight junction proteins, and lowering levels of HMGB1, TLR4, and MyD88. Molecular docking showed strong binding energies between butyrate and HMGB1 (-3.7 kcal/mol) and TLR4 (-3.8 kcal/mol).

Conclusions: According to bioinformatics predictions, butyrate mitigates IRI via multiple-target and multiple-channel mechanisms. The extent of IRI can be reduced by butyrate through the inhibition of the HMGB1-TLR4-MyD88 signaling pathway, which is related to senescence.

## INTRODUCTION

Intestinal ischemia-reperfusion (IR) is a common clinical condition characterized by a complex mechanism and various therapeutic approaches [1]. It can be categorized into acute and chronic types. Acute intestinal ischemia-reperfusion often results from significant blood loss, cardiac arrest, or severe infection, while chronic intestinal ischemia-reperfusion mainly arises from atherosclerosis [2]. The onset of intestinal ischemia-reperfusion stimulates a cascade of harmful responses, entailing compromised intestinal mucosal barrier function, disrupted intestinal microcirculation, and oxidative stress [3]. These reactions can lead to serious outcomes like ischemia, hypoxia of the intestinal tissue, necrosis, and bleeding. Hence, exploring strategies to alleviate IR is critically important.

Aging individuals are more prone to ischemia-reperfusion injury, potentially due to the presence of coexisting conditions such as hypertension, diabetes, and obesity [4, 5]. However, age itself is intrinsically linked to the cardiovascular system as an independent risk factor, making prevention and treatment of IR from a senescence perspective worth discussing.

Current evidence suggests that butyrate has protective effects against intestinal ischemia-reperfusion injury (IRI) [6]. In addition to its role in regulating oxygen free radical metabolism, butyrate also eases damage to the intestinal mucosal barrier and improves intestinal microcirculation [7]. Multiple studies have suggested that butyrate effectively reduces the oxidative stress response induced by intestinal ischemia-reperfusion, enhances the functionality of the intestinal mucosal barrier, and minimizes injury and necrosis in intestinal tissue cells [6, 7]. Butyrate may also increase regional blood flow and microcirculation and increase the critical expression of intestinal vascular endothelial growth factor, thereby augmenting tissue oxygenation following intestinal ischemia and reperfusion [8, 9].

However, debates persist regarding the exact mechanism of butyric acid in the context of intestinal ischemia-reperfusion. However, larger clinical trials are needed to validate its safety and effectiveness and provide comprehensive guidelines for its use. It has been demonstrated that intestinal ischemia-reperfusion activates Toll-like receptor 4 (TLR4), which binds to its ligand, triggering downstream signal transduction regulated by MyD88 [10, 11]. This cascade then activates NF-κB p65 and the transcription of downstream genes, coding inflammatory mediators, adhesion molecules, and apoptosis regulators, contributing to the inflammatory response, apoptosis, and local tissue damage [12, 13]. Therefore, inhibition of the TLR4 signaling pathway could mitigate the negative effects of IRI [14]. This research investigated whether butyrate treatment affects TLR4 signaling in this disease model.

## MATERIALS AND METHODS

### Analyzing network pharmacological data

The molecular structure file of butyrate was obtained from PubChem (https://pubchem.ncbi.nlm.nih.gov/) and subsequently uploaded to the target prediction tool of the Bioinformatics Analysis Tool for Molecular mechANism of Traditional Chinese Medicine (Batman-TCM) database for the purpose of identifying potential targets associated with butyrate [15]. To thoroughly investigate the potential drug targets of butyrate, the screening process employed an unrestricted Score cutoff condition. The GeneCards database (https://www.genecards.org/, GeneCards Version 5.16) was utilized to search for reported genes associated with aging and IRI, using the keywords “intestinal ischemia-reperfusion injury” and “senescence” [16].

### Analysis of transcriptomes in single cells

The single-cell RNA sequencing (scRNA-seq) dataset GSE190581 was downloaded from the Gene Expression Omnibus (GEO) platform (https://www.ncbi.nlm.nih.gov/geo/) [17]. The detection of this data set was based on 10X genomics platform, and two intestinal tissues damaged by intestinal ischemia-reperfusion in mice were detected. For the data set GSE190581, the “seruat” package is used for scRNA-seq data analysis. The specific process of its analysis was as follows: 1) The PercentFeatureSet function was used to determine the proportion of mitochondrial genes, and correlation analysis was used to study the relationship between sequencing depth and mitochondrial gene sequence and/or intracellular total sequence. 2) Set each gene to be expressed in at least 3 cells and 200 genes. 3) The number of genes expressed in each cell was more than 300 and less than 5000, the mitochondrial content was less than 10%, and the UMI of each cell was at least 1000. 4) Data are filtered and then standardized, features are selected and normalized, batch removals are performed, and dimensionality reduction clustering is performed. 5) Annotations for cells were taken from CellMarker 2.0 (http://bio-bigdata.hrbmu.edu.cn/CellMarker/). 6) After that, FindAllMarkers was used to identify genes whose expression differed between different cells. The set filter condition for the log fold change (FC) was 0.25 [18].

### Construction and analysis of the butyrate-IRI target network

First, the mouse genes from single-cell transcriptomics were transformed into human genes via the msigdbr package. Then, based on the four genes related to aging and intestinal ischemia-reperfusion injury identified in the GeneCards database, the differentially expressed genes were identified via scRNA-seq analysis, and the genes of potential drug targets were subjected to Venn diagram screening. Finally, the drug action and expected IRI targets were identified. The common targets were loaded into the STRING database, the species was limited to “*Homo sapiens*”, a protein-protein interaction (PPI) network diagram was created, the required minimum interaction score was set to a high confidence level of 0.07, and the discrete targets were hidden. Then, the PPI network diagram was analyzed by using Cytoscape (version 3.7.0) with the MCODE tool to analyze the targets of key nodes.

### GO and KEGG analysis

To identify the biological functions of butyrate and IRI-related target genes in gene ontology (GO) and Kyoto Encyclopedia of Genes and Genomes (KEGG) pathway enrichment analysis, clusterProfiler, org was used for enrichment analysis [19]. Then the analysis adopts the org.Hs.eg.db, enrichplot and ggplot2 packages, and the critical value standard was *P*< 0.05 [20].

### Mouse model of intestinal ischemia-reperfusion injury

Thirty male C57BL/6 mice, approximately 8 weeks old, were randomly divided into 6 groups from Liaoning Changsheng Biotechnology Co., Ltd. (animal batch number: SCXK (Liao) 2020-0001), that is, group A was the normal group (normal feeding); group B was sham operation group (sham); group C was the model group (modeling); group D was the control group (modeling+equal volume PBS); group E was butyrate group 1 (modeling +100 mg/kg butyric acid); and group F was butyrate group 2 (modeling +300 mg/kg butyric acid). Mice lived in a fixed circadian rhythm and were kept in separate cages in a temperature-controlled room, and they were allowed free access to food and water. The mouse experiment in this study was approved by the Ethics Committee of The Fourth Hospital of Hebei Medical University (2021022). An intestinal I/R injury model was generated as described above [21]. The normal group was raised routinely without any modeling. In the model, control, and butyric acid groups, mice were anesthetized with 1% pentobarbital sodium, and the right abdominal cavity was exposed. The superior mesenteric artery (SMA) was located approximately 2 mm below the dark region of the right abdomen. We timed the clamping of the SMA then sutured in layers using 4-0 silk thread. After 45 minutes, we removed the sutures and the arterial clamp. In the sham operation group, the SMA was not clamped, but the same operation procedures were carried out. Immediately after resuscitation, the mice underwent further treatments: the butyrate groups received injections of either 100 mg/kg or 300 mg/kg butyrate via the SMA, whereas the vehicle group received an injection of the same volume of PBS via the SMA. The normal group and the model group received no injections. During the entire operation, the body temperature of the mice was maintained at 37° C using a constant temperature blanket. Mice were euthanized at 30 min, 24 h, and 72 h post-reperfusion, and small intestine samples were collected. Each sample was divided into three sections: two were treated with either 2% paraformaldehyde or 2.5% glutaraldehyde, and the remaining section was dried on filter paper and stored at -80° C for testing.

### Transmission electron microscopy

Colon cells were observed for their structural state using transmission electron microscopy (TEM). Fixation of colon tissue with glutaraldehyde 2.5% was followed by three washes in 0.1 M PBS. Infiltrated and buried tissue was first fixed with 1% osmium tetroxide for two hours, followed by dehydration with 30%, 50%, 70%, 90%, 95%, and 100% ethanol. Afterwards, 60 nanometer-thin slices of colon tissue were cut. After avoiding carbon dioxide through the use of 2.6% lead citrate solution, the staining effect of colon cells was observed by TEM (JEM-1200EX, JEOL, Tokyo, Japan).

### Hematoxylin and eosin staining

Hematoxylin and eosin (H&E) staining was performed on 5-micron paraffin coronal sections of mouse small intestine fixed for 48 hours with 2% paraformaldehyde [22]. Glass slide was magnified by 100 times under an optical microscope (Nikon, Tokyo, Japan).

### Immunohistochemical analysis

A 4 mm section of paraffin-embedded tissue was cut into 4 pieces. The chips were dewaxed in xylene and dehydrated in ethanol, after which the sections were repaired with citrate buffer antigen. Following incubation with 3% hydrogen peroxide for 10 minutes, the slices were rinsed three times with PBS to inhibit endogenous peroxidase activity. A block of 3% BSA was applied to the slices, which were then incubated at 4° C overnight with the primary antibody. Slices were incubated for an hour at room temperature with the second antibody conjugated with horseradish peroxidase (HRP). The concentrations of both Occludin and Claudin-1 were 1:200 (Wanleibio, Shenyang, China). IHC was performed with a DAB chromogen kit, and images were taken with a Nikon NIS element BR optical microscope (Nikon, Tokyo, Japan). ImageJ software can be used to analyze all the IHC images. All positive and negative cells in each visual field were counted, and the percentage of all positive cells was calculated.

### Western blotting

Proteins were extracted from abdominal tissue by lysing it in RIPA lysis buffer and benzyl sulfonyl fluoride (PMSF) for 5 minutes on ice. Following manufacturer’s instructions (Beyotime, China), cytoplasmic proteins and nucleic acids are extracted using nuclear and cytoplasmic protein extraction kits, respectively. An equal amount of protein lysate (30 g per lane) was separated by SDS-PAGE on 10% polyacrylamide gel and transferred to polyvinylidene fluoride membranes. Overnight at 4° C, we used the primary antibodies HMGB1 (cat. no. WL03023, diluted at 1:500, Wanleibio), TLR4 (cat. no. WL00196, diluted at 1:500, Wanleibio), and MyD88 (cat. no. WL02494, diluted at 1:500, Wanleibio). After washing the membrane four times with TBST, the membrane was incubated with the secondary antibody for four hours at 4° C the next day. To quantify the strip strength, Quantity One (Bio-Rad, Shanghai, China) was used as a reference. We standardized the relative protein levels to the same concentration used for the control group.

### Molecular docking

The search conditions for UniProt (https://www.uniprot.org/) were limited to humans, and the numbers for HMGB1 and TLR4 were P09429 and O00206, respectively. Next, the protein number in the RCSB PDB (https://www.rcsb.org/) was entered to download the corresponding structure. Using PyMOL 2.4.1, crystal water from proteins was removed, and small molecules from the PubChem database were subsequently imported into ChemBio3D Ultra 14.0 software to minimize their energy. Using CB-Dock (http://clab.labshare.cn/cb-dock/), the binding site was located automatically, the center and size were calculated, the docking box size was customized to suit the query ligand, and then AutoDock Vina was used for molecular docking. To preliminarily assess the binding activity of compounds to targets, Vina scores were calculated, where lower values indicate more stable binding.

### Statistical analysis

The mean and standard deviation of the data are expressed as the means of two groups. When the variance was equal, one-way analysis of variance (ANOVA) was used to compare the means of the groups, and the Dunnett test was used to confirm the equality of the variance. Instead, Brown-Forsyth tests are carried out without assuming equal variances. Repeated measures ANOVA was used to calculate the covariance of the measured data if they conformed to a normal distribution. The data were analyzed using GraphPad Prism 8.0 and R software (version 4.2.2; https://www.r-project.org/). *P* <0.05 was considered to indicate statistical significance in this study.

## RESULTS

### Target prediction

Butyrate was obtained from PubChem with a PubChem CID of 264. According to Batman-TCM, under conditions where the Adjusted *P*-value is less than 0.05, 16,382 predicted drug targets were identified (Supplementary Table 1). A total of 2766 and 6275 related genes were screened in GeneCards based on intestinal ischemia-reperfusion injury (Supplementary Table 2) and senescence (Supplementary Table 3), respectively.

### Analysis of scRNA-seq

**Figure 1:**
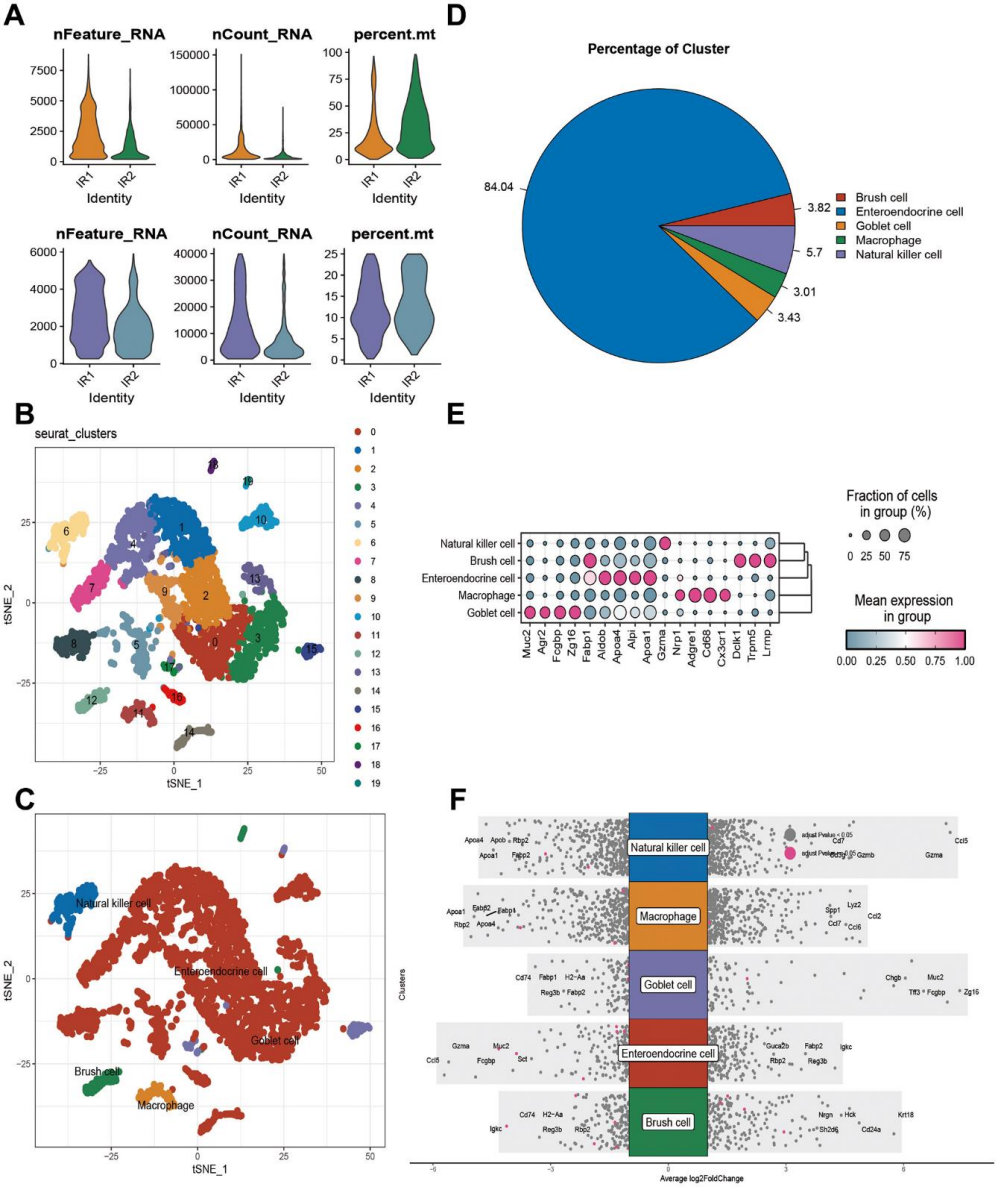
**Results of single-cell analysis of the GSE190581 dataset.** (**A**) Number of cells before and after quality control. (**B**) Results of subset analysis. (**C**) Cell annotation results. (**D**) Percentage of five cell names in GSE190581. (**E**) Marker expression in five cell types. (**F**) Differential gene expression between cells.

**Figure 2:**
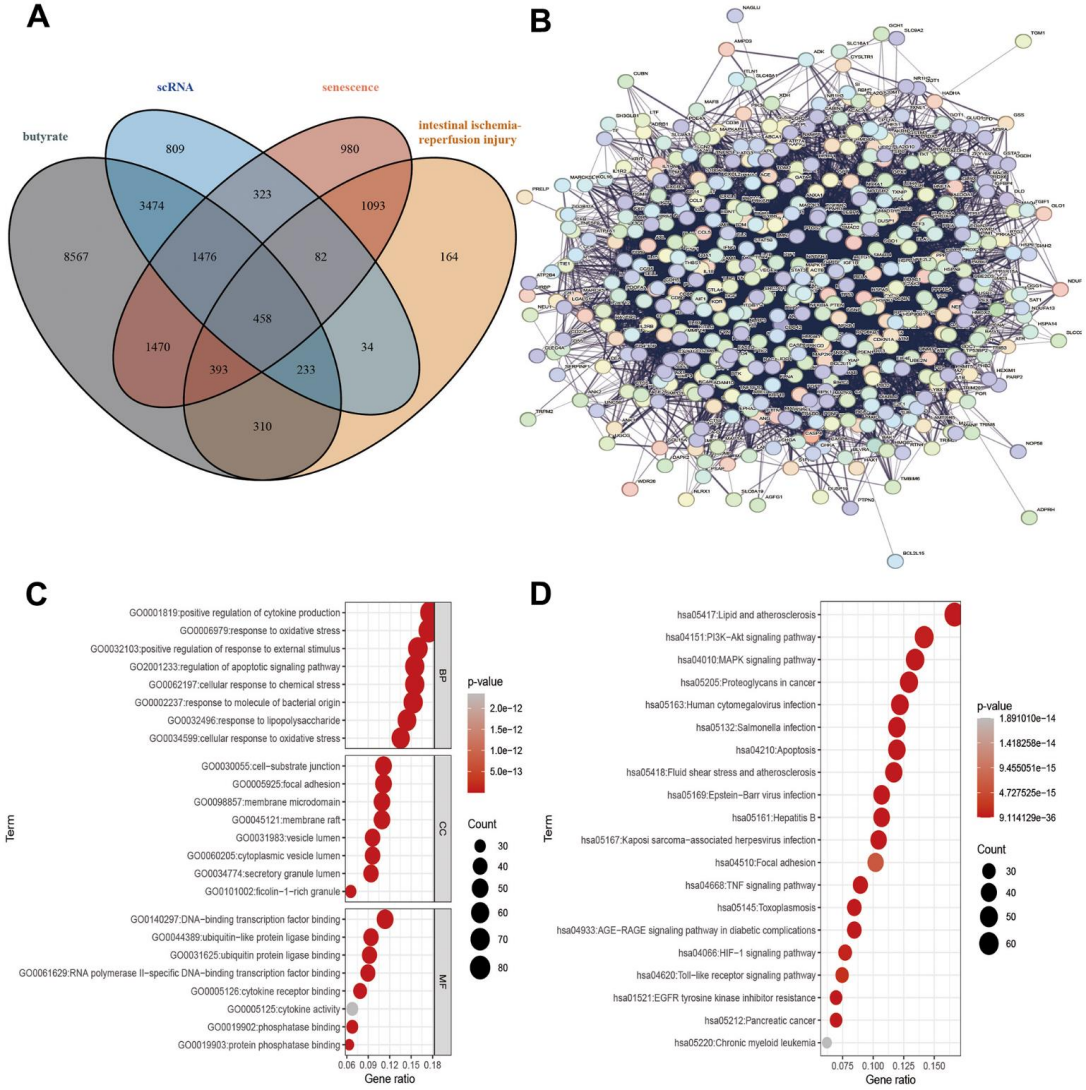
**Target gene screening against IRI, PPI of target genes and, functional enrichment analysis of target genes.** (**A**) Interacting genes, (**B**) PPIs, (**C**) GO analysis, (**D**) KEGG analysis.

When conditional screening was applied to the single cell data set GSE190581, 4086 cells were retained (Figure 1A). As a result of setting resolution to 0.3, 20 cell subsets were obtained, and the different kinds of cells were annotated as well as the varying numbers and proportions of the cell types (Figure 1B–1D). There are five types of cells: Goblet cell (Muc2, Agr2, Fcgbp, Zg16), Enteroendocrine cell (Fabp1, Aldob, Apoa4, Alpi, Apoa1), Natural killer cell (Gzma), Regulatory T (Nrp1), Macrophage (Adgre1, Cd68, Cx3cr1), and Brush cell (Dclk1, Trpm5, Lrmp) (Figure 1E, 1F). A total of 6889 differentially expressed genes (Supplementary Table 4) were identified between different cells in the data set using Log FC = 0.25 (Figure 2A).

### Targets intersecting

The following four parts of genes were intersected: the target gene of butyrate structure prediction, the genes related to intestinal ischemia-reperfusion injury and senescence in GeneCards, and the DEGs in the single-cell dataset GSE190581. In this study, a total of 458 potential target genes were ultimately identified (Figure 2A).

### PPIs

In the STRING database, 458 potential target genes were used to construct protein interaction networks. In this network, there are 458 nodes and 10628 edges (Figure 2B). The key genes in the above networks were analyzed using the MCODE plug-in in Cytoscape. As a result, a key gene network diagram comprising 77 nodes and 1078 edges was constructed (Supplementary Figure 1).

### Functional annotation and pathway enrichment analysis

Functional enrichment analysis was performed on 458 genes in the intersection genes. The biological process (BP) enrichment items include positive regulation of cytokine production, response to oxidative stress and regulation of apoptotic signaling pathway; the cellular component (CC) enrichment items include cell-substrate junction, focal adhesion and membrane microdomain; the molecular function (MF) enrichment items include DNA-binding transcription factor binding, ubiquitin-like protein ligase binding and ubiquitin protein ligase binding (Figure 2C). In addition, KEGG enrichment analysis reveals PI3K-Akt signaling pathway, MAPK signaling pathway, and Toll-like receptor signaling pathway (Figure 2D).

### Intestinal tissue subjected to TEM

**Figure 3:**
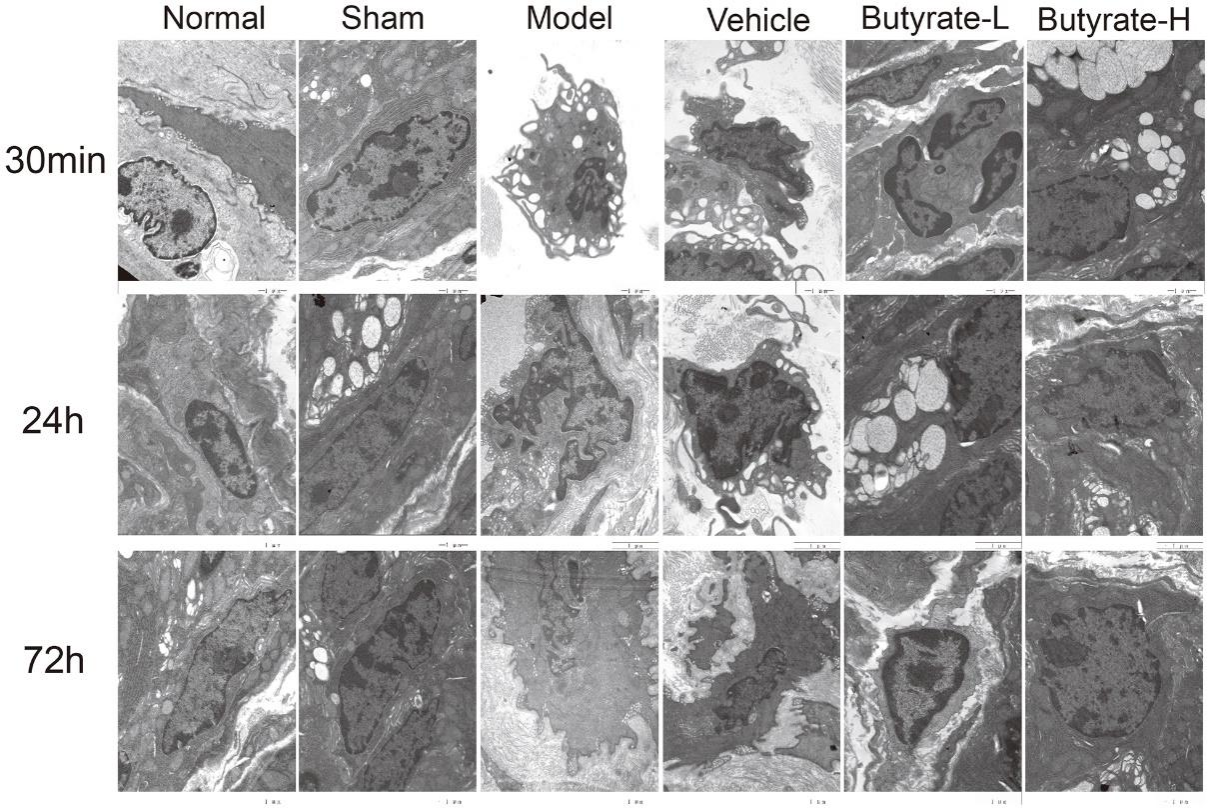
The results of transmission electron microscopy of intestinal tissue.

Figure 3 shows that the interstitial cells of normal Cajal were found in the normal and sham groups of stained intestinal tissue. Both groups contain many intracellular organelles with complete morphology, such as mitochondria and the Golgi apparatus. Interstitial cells of Cajal were protuberant in the model group, and mitochondria shriveled, cristae and some cytoplasm were lacking. Additionally, Cajal’s interstitial cells lost contact with intestinal nerves. The butyrate concentration increased with increasing sodium butyrate concentration, reducing damage from both high and low doses of butyrate.

### H&E staining

**Figure 4:**
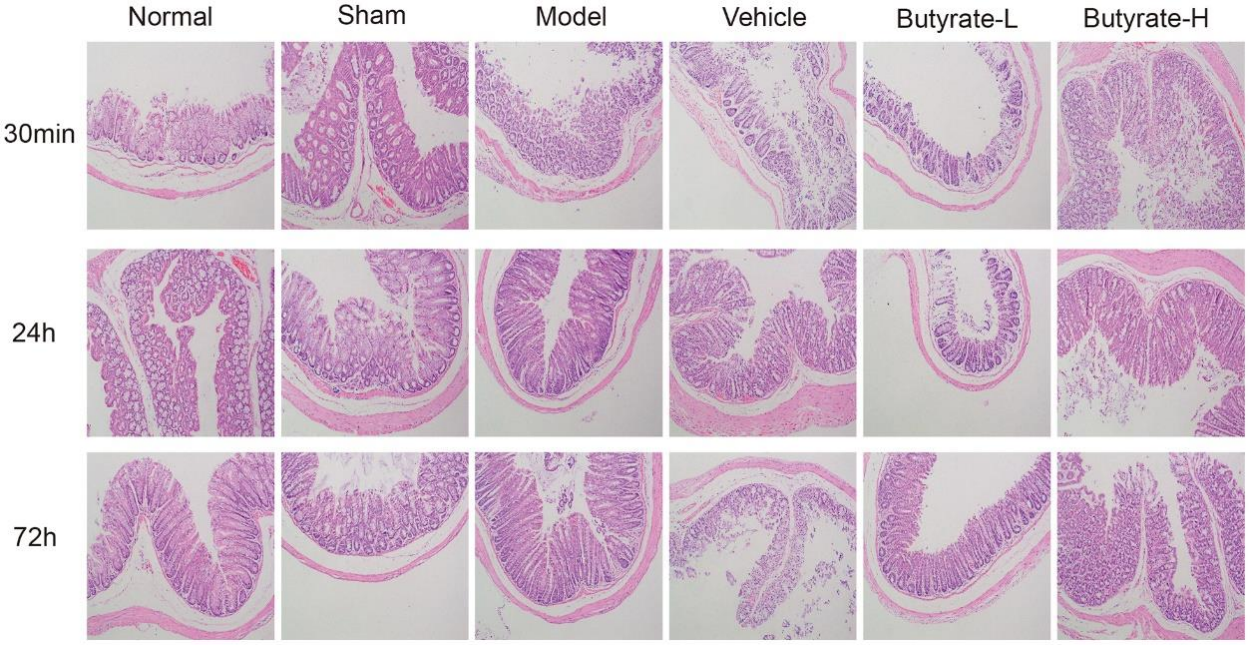
Hematoxylin and eosin staining in this study.

H&E staining revealed that the intestinal folds in the normal group were circular, semicircular, or spiral and that the mucosal surface was covered with numerous small intestinal villi (Figure 4). The results in the sham operation group were similar to those in the normal group. In the model and vehicle groups, there were scattered epithelial structures in the intestinal villi, cells detached at the tips of the villi, and noticeable gaps formed. The morphological success of the model was evident. Increasing the dosage of butyrate improved the epithelial structure of the intestinal villi, but shedding of the epithelial layer and desquamation of the epithelium and villi still occurred.

### IHC analysis

**Figure 5:**
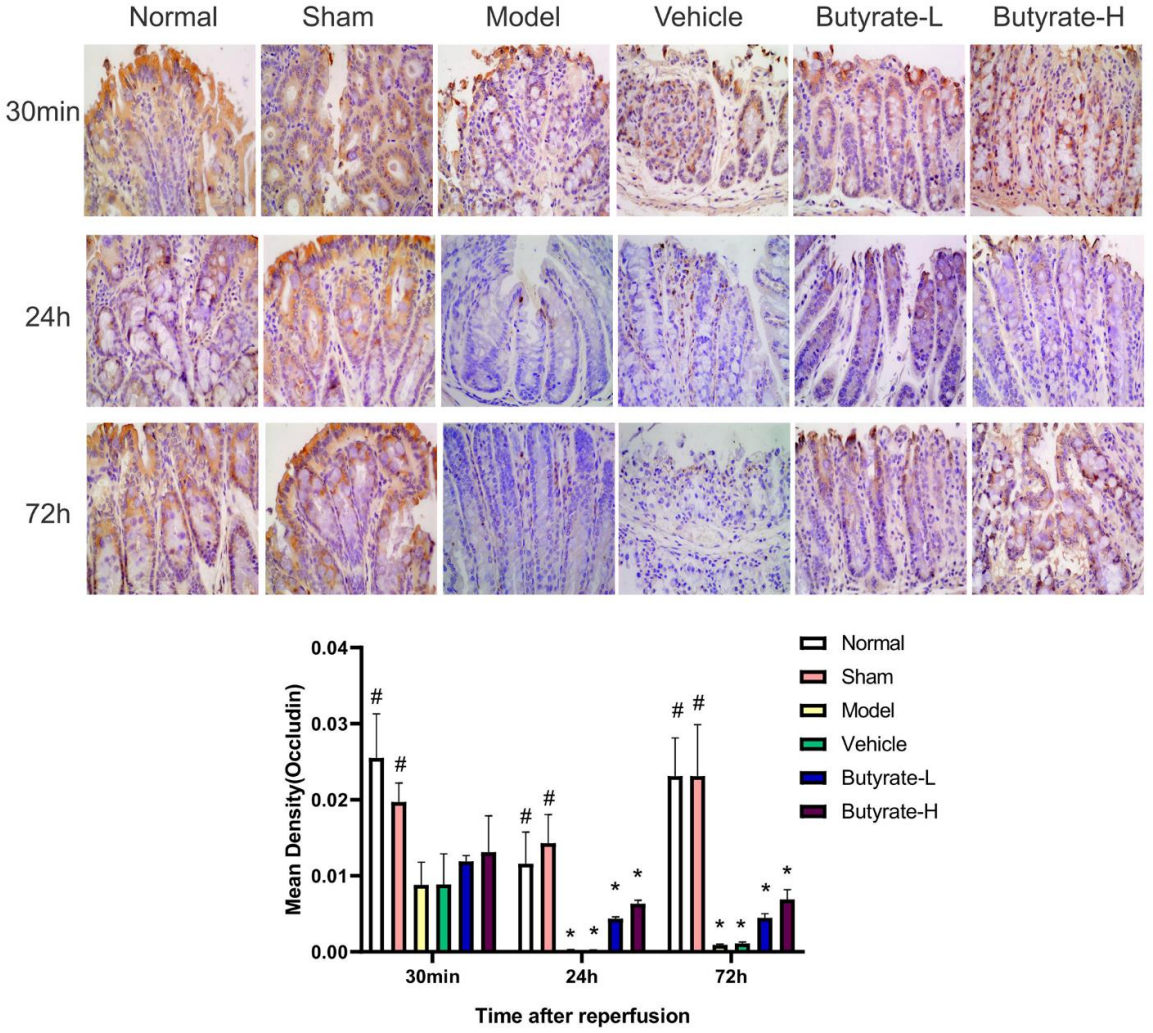
**The immunohistochemical analysis of Occludin.** It represents a group that differs significantly from the vehicle group with the symbol (#). It represents a group that differs significantly from the vehicle group with the symbol (*).

IHC analysis of Occludin is shown in Figure 5. The normal and sham groups exhibited Occludin protein expression in the intestinal mucosa, which was evenly distributed and prominent in the cell membrane. After 24 hours, the Occludin protein levels decreased in the model and vehicle groups, and their distributions became uneven over time. As the dose of butyrate was increased, Occludin protein expression decreased in comparison to the vehicle group as well as the model group. There was a difference between the Occludin levels in the butyrate groups and those in the sham group at 24 and 72 hours (*P*<0.05).

**Figure 6:**
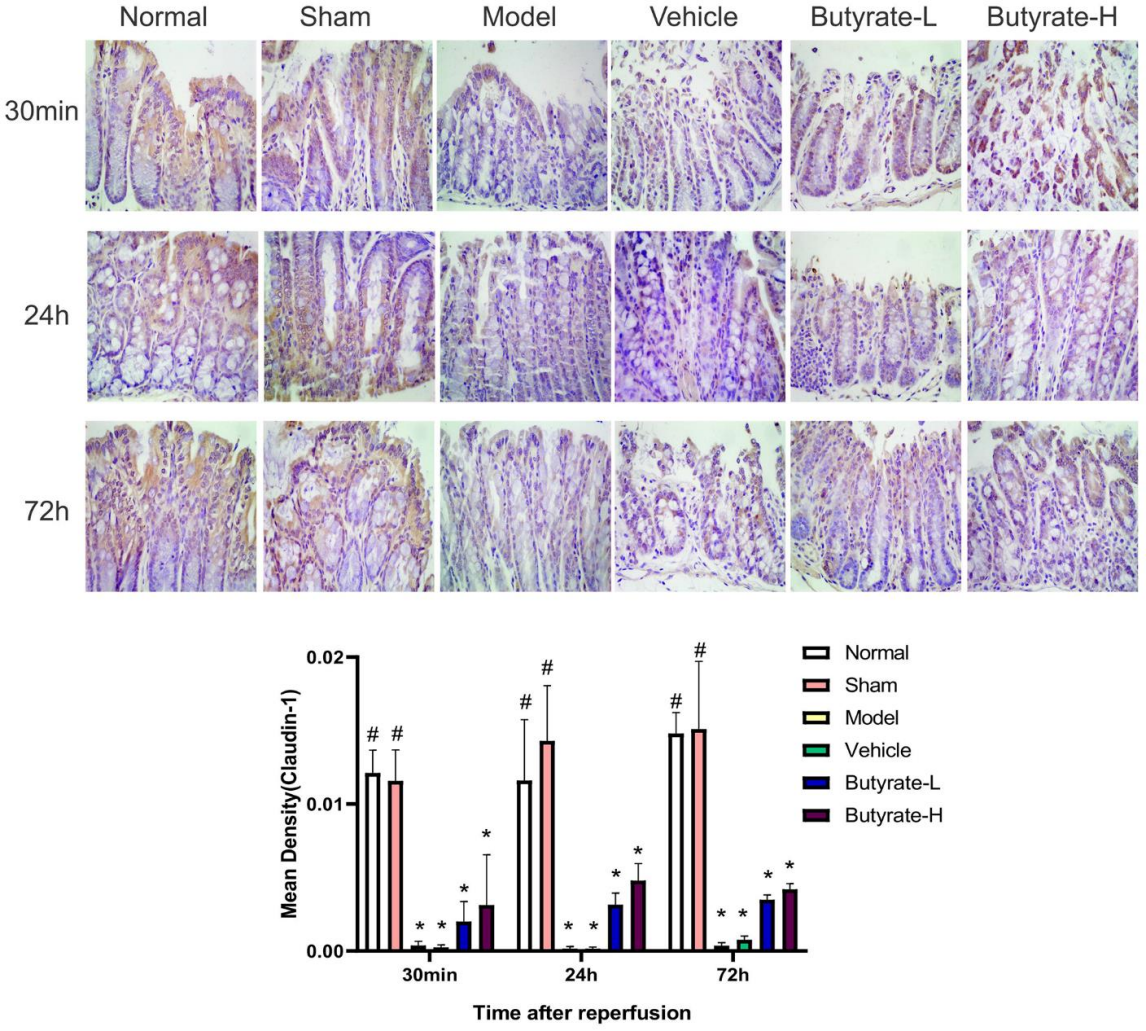
**The immunohistochemical analysis of Claudin-1.** It represents a group that differs significantly from the vehicle group with the symbol (#). It represents a group that differs significantly from the vehicle group with the symbol (*).

Figure 6 shows the results of IHC staining for Claudin-1. Claudin-1 was clearly distributed on the cell membrane in the intestinal mucosa of the normal and sham groups. A decrease in Claudin-1 expression was observed in the model group and vehicle group, and an uneven distribution of the protein was also observed. Claudin-1 protein expression increased over time in response to increasing butyrate concentrations. Compared with those in the sham group, the Claudin-1 levels in the two groups were different after butyrate treatment (*P*<0.05).

### Western blotting

**Figure 7:**
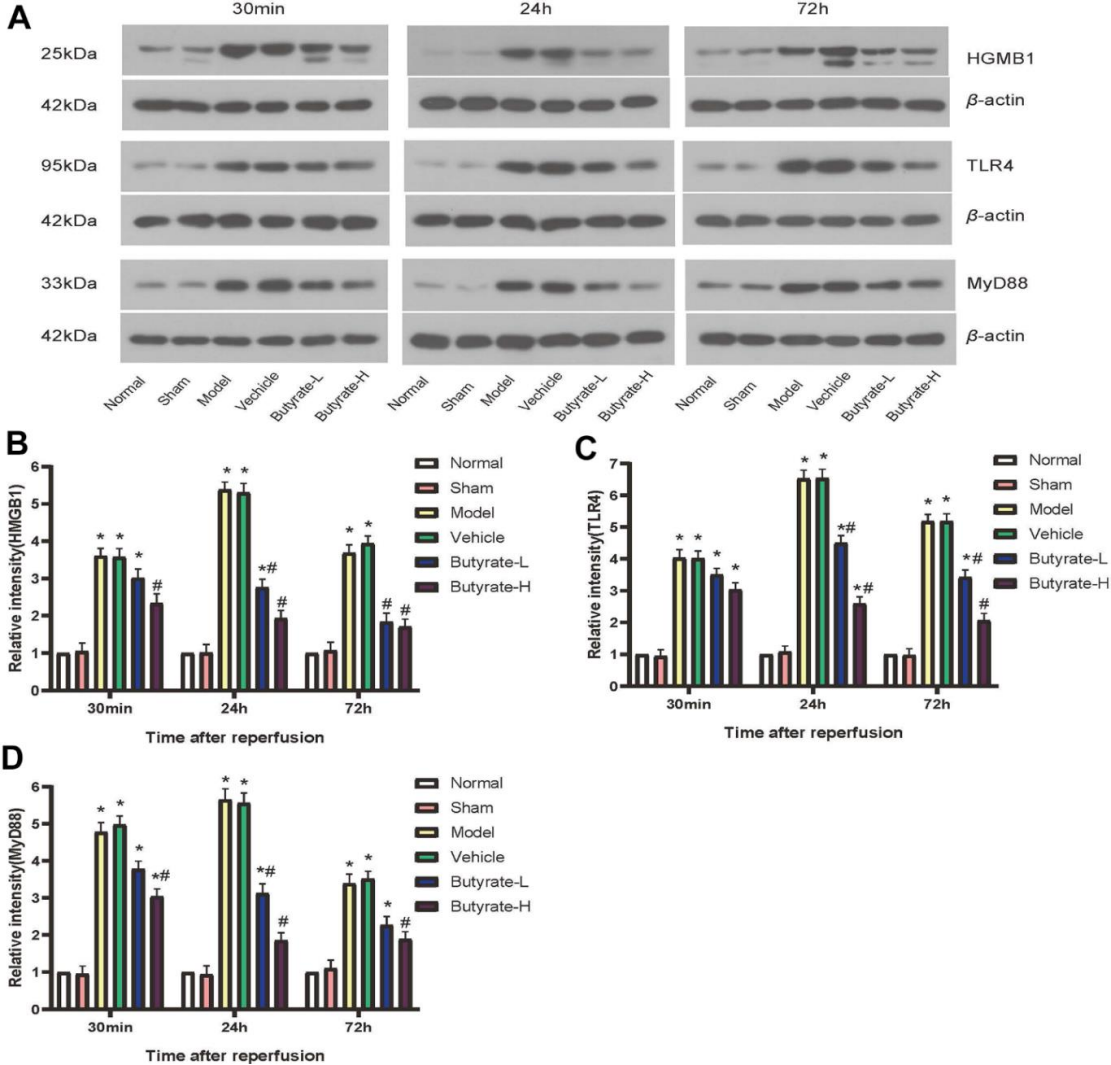
**Butyrate may protect against IRI via the HMGB1-TLR4-MyD88 signaling pathway.** (**A**) Western blotting results of three proteins in different groups. (**B**) Comparison of Western blotting results of HMGB1 in different groups. (**C**) Comparison of Western blotting results of TLR4 in different groups. (**D**) Comparison of Western blotting results of MyD88 in different groups. It represents a group that differs significantly from the vehicle group with the symbol (#). It represents a group that differs significantly from the vehicle group with the symbol (*).

Figure 7 illustrates the varying expression levels of HMGB1, TLR4, and MyD88 across each group in this study. It was observed that the levels of HMGB1 protein began to rise at different times post induction of the IRI model in mice, with the specific timing varying according to the duration. A statistically significant difference (*P*<0.05) was found in HMGB1 protein expression between the model group and the vehicle group when compared to the sham group. High-dose butyrate intervention led to a decrease in HMGB1 protein expression after 30 minutes, which differed significantly from the vehicle group (*P*<0.05). This study reveals that butyrate intervention is concentration-dependent; higher concentrations of butyrate result in a time-dependent decrease in HMGB1 expression levels. Unique to this study is the observation that TLR4 and MyD88 proteins are expressed at similar levels as HMGB1. The expression levels of TLR4 and MyD88 proteins increased differently following both the model and the vehicle interventions. However, these expression levels decreased following butyrate intervention. A statistically significant difference (*P*<0.05) was noted between high-dose butyrate and vehicle groups concerning the expression of TLR4 and MyD88 proteins.

### Molecular docking

**Figure 8:**
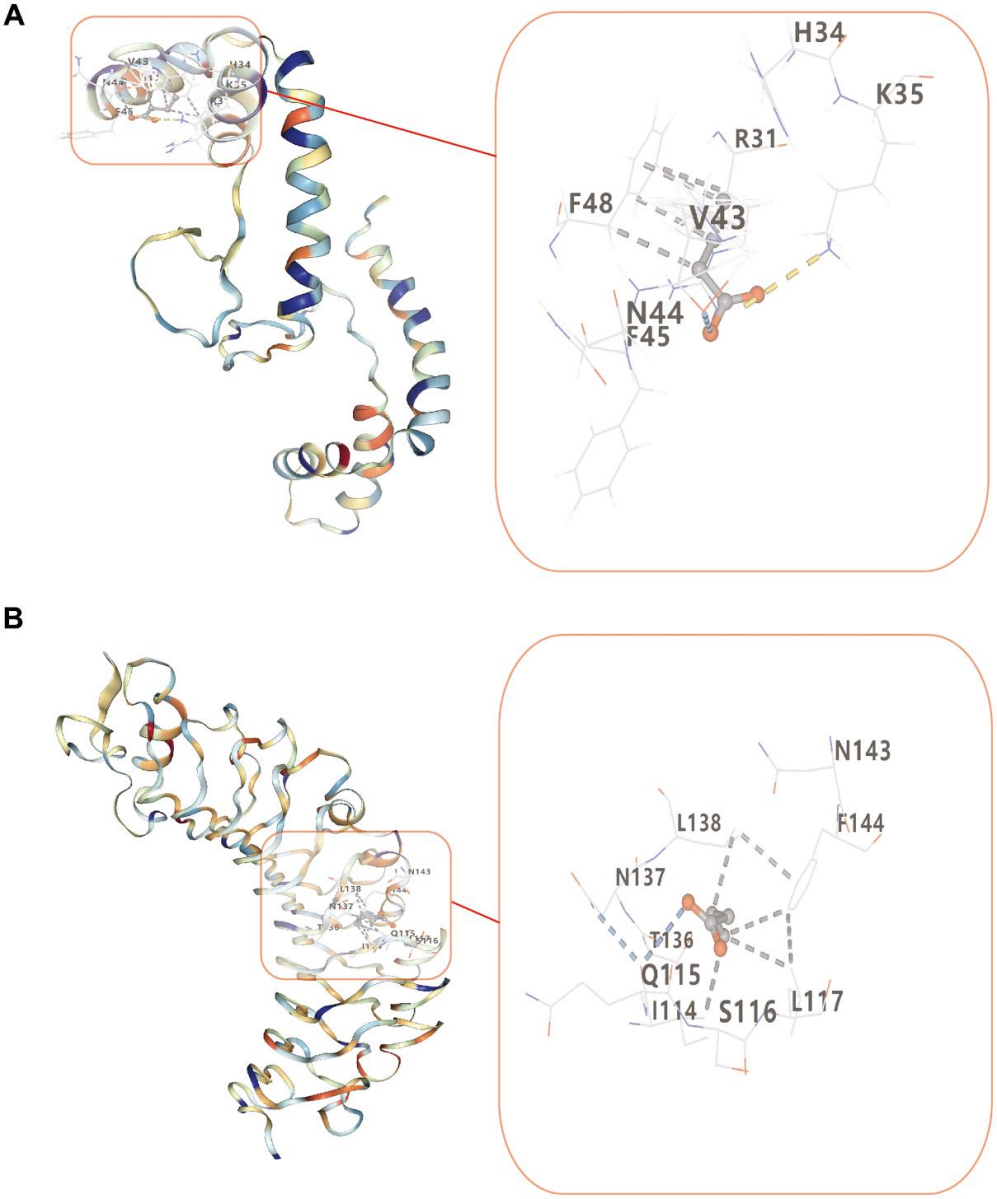
**Molecular docking of butyrate and two target proteins.** (**A**) Molecular docking between butyrate and HMGB1. (**B**) Molecular docking between butyrate and TLR4.

Figure 8 illustrated how lower Vina scores indicated stronger and more stable interactions between the compound and the receptor. HMGB1 and TLR4 binding strengths of butyrate were -3.7 kcal/mol and -3.8 kcal/mol, respectively (Table 1). This indicated HMGB1 and TLR4 had a certain affinity for butyrate.

**Table 1:** Molecular docking results of the targets and butyrate.

**Molecule name**	**Target name**	**PDB ID**	**Score (kcal/mol)**	**Cavity size**	**Centre x**	**Centre y**	**Centre z**	**Size x**	**Size y**	**Size z**
butyrate	HMGB1	2YRQ	-3.7	114	-25	-21	2	15	15	15
	TLR4	2Z62	-3.8	154	17	-14	8	15	15	15

## DISCUSSION

People suffering from ischemic bowel disease are usually middle-aged or elderly, with severe cases having intestinal obstruction [23]. Because the colon has a relatively slow blood flow and poor microcirculation system and is more sensitive to autonomic nerve stimulation than the small intestine is, the colon is more likely to suffer from ischemia [24]. Colon hypoperfusion results in a reduction in colon blood flow, which impairs the metabolic function of colon cells and leads to acidosis, dysfunction, and eventual cell death [24, 25]. Microvascular ischemia-reperfusion in the colon is responsible for this pathophysiological mechanism. Additionally, elderly patients are susceptible to IRI injury, which is associated with their own basic diseases (arteriosclerosis, cerebral infarction, coronary heart disease, etc.) [4, 26–28]. The study’s enrichment analysis shows that aging is closely linked to cytokine production and oxidative stress. Aging is associated with increased inflammatory activity, as evidenced by higher levels of TNF-α, IL-6, cytokine antagonists, and acute phase proteins in the body [29]. Oxidative stress can also lead to the secretion of inflammatory cytokines [30]. KEGG analysis indicates that TLRs signaling pathway plays a role in inflammation in both normal aging and diseases with cognitive decline [31]. Many drugs targeting anti-aging focus on the PI3K-Akt [32, 33] and MAPK signaling pathways [34, 35]. It is therefore extremely important to reduce intestinal IRI with drugs.

The collection of senescence-related genes and the correlation between IRI target genes and butyrate have led to the KEGG analysis indicating that butyrate is intimately associated with signal pathways connected to inflammation and antioxidants. The PI3K-Akt signaling pathway, an important pathway for maintaining the body’s oxidative homeostasis [36] was highlighted. Evidence suggests that liraglutide may reduce inflammatory reactions and tissue apoptosis in the gut, and also protect intestinal immune responses via NF-κB and PI3K-Akt pathways [21]. After treatment with Ellagic acid, transcriptome analysis revealed that AKT1-specific mRNAs were predominantly located in PI3K-AKT signaling pathways in IRI mice [37]. Additionally, an oxygen-glucose deprivation/reoxygenation (OGD/R) model was developed to simulate intestinal IRI. Activating the PI3K-Akt-p53 signaling pathway, adenosine A1 receptor agonists could decrease OGD/R damage in Caco-2 cells, possibly due to their anti-apoptotic effect [38]. Both *in vitro* and *in vivo* studies showed a link between intestinal IRI and p38 MAPK activation [39], consistent with our bioinformatics research findings.

Propofol, a p38 MAPK-NF-κB signaling pathway inhibitor, can lessen intestinal edema and inflammation, thereby mitigating and treating intestinal IRI in rats [40]. Furthermore, 6-gingerol inhibited p38 MAPK activation and ROS formation in an ischemia-reperfusion model of rat intestinal ischemia [41]. Toll-like receptor signaling also plays a crucial role in the IRI process. Toll-like receptors (TLRs), known as microbial sensors due to their transmembrane nature from previous studies, are capable of coordinating the body’s defense against infection and detecting dead cell products in host tissue. As one of the most representative tissue injury states, IRI is likely unavoidably linked to a TLR-mediated molecular mechanism [42]. TLR2 has been implicated in inducing inflammatory mediators, such as TNF-a, causing chronic damage to the small intestine during adult IRI [43]. Besides sensing and defense functions, TLRs also play a significant role in wound repair. For instance, the dsRNA-sensing receptor (TLR3) is involved in signal transduction and rescue of tissue damaged by inflammation [44]. The primary focus of this study is the potential involvement of TLR4 in various IRI-induced inflammatory processes [45]. The TLR4 receptor plays a key role in the production of prostaglandin E2 (PGE2) upon intestinal ischemia and reperfusion induced by Cox-2 [46], and under macrophage stimulation, TLR4 also induces the secretion of various cytokines [47]. In rats with intestinal ischemia-reperfusion injuries and OGD ischemia-reperfusion injuries, dexamethasone alleviated the injury. This protective effect may be associated with anti-inflammatory effects and the inhibition of TLR4-MyD88-NF-κB signaling pathways [48]. The Changqing mixture is believed to prevent IRI through the TLR4-NF-κB pathway [49]. All these studies indicate the crucial role of TLR4 in regulating IRI.

Some articles have reported that butyrate exhibits therapeutic effects on ischemia-reperfusion injury (IRI), as well as detailing its molecular mechanisms. Butyrate intervention improved intestinal injury in a rat model of IRI and reduced the levels of inflammatory factors and leukocyte infiltration. Moreover, butyrate aids in maintaining intestinal barrier integrity by increasing the expression of tight junction proteins and minimizing endotoxin translocation [6, 8, 50, 51]. In rats with IRI induced by renal transplantation, butyrate amplified intracellular oxidative stress and inflammation to mitigate IRI [51]. However, the precise mechanism through which sodium butyrate impacts IRI remains unclear. Bioinformatics and animal experiments conducted in this study confirmed that sodium butyrate did not delineate signals related to HMGB1-TLR4 and IRI target genes. In mice models with IRI, injections of anti-HMGB1 and anti-MyD88 inhibited the expression of HMGB1 and MyD88, reducing serum inflammatory cytokines [52]. This antibody injection also decreased lung and small intestine tissue damage in the intestinal IRI mouse model compared to the control group. Consistent with the results from the previous study, post-butyrate intervention, levels of HMGB1 and MyD88 in IRI were found to be lower than those in the model group. Neonatal mouse models of necrotizing enterocolitis exhibited reduced levels of HMGB1, TLR4, and inflammatory cytokines after butyrate pretreatment [7]. Moreover, the interplay between HMGB1-TLR4-MyD88 and ischemia-reperfusion signaling pathways has been studied [53, 54]. Propofol pretreatment alleviated IRI-induced lung injury in pigs by inhibiting the HMGB1-TLR4-PKR signaling pathway [55]. Given the interactions among HMGB1, TLR4, and MyD88 identified in previous research, this study analyzed the effects of butyrate intervention on their proteins. The findings indicated that butyrate also reduced intestinal damage after IRI through the HMGB1-TLR4- MyD88 signaling pathway.

Due to time and energy constraints, we only studied butyrate’s effect on the HMGB1-TLR4-MyD88 signal pathway in IRI. Our research is limited to *in vitro* animal experiments and requires more experiments and clinical validation. Additionally, the small sample size of single cell data used and the information of other public data which was limited in this study highlight the need for more data collection in the future. Further experiments are necessary to support our research.

## CONCLUSIONS

IRI can damage intestinal tissues and trigger inflammatory response and oxidative stress, but butyrate can effectively counteract these effects. Furthermore, butyrate inhibits the HMGB1-TLR4-MyD88 signaling pathway in intestinal tissue, which is beneficial for preventing and treating IRI-induced intestinal tissue injury.

## Supplementary Material

Supplementary Figure 1

Supplementary Table 1

Supplementary Table 2

Supplementary Table 3

Supplementary Table 4
