# Supplementary figures and images for: Effects of butyrate on intestinal ischemia-reperfusion injury via the HMGB1-TLR4-MyD88 signaling pathway
Source: Aging (Albany NY). 2024 May 3;16(9):7961–78. doi: 10.18632/aging.205797 (PMC11131991; doi:10.18632/aging.205797)

SUPPLEMENTARY FIGURE

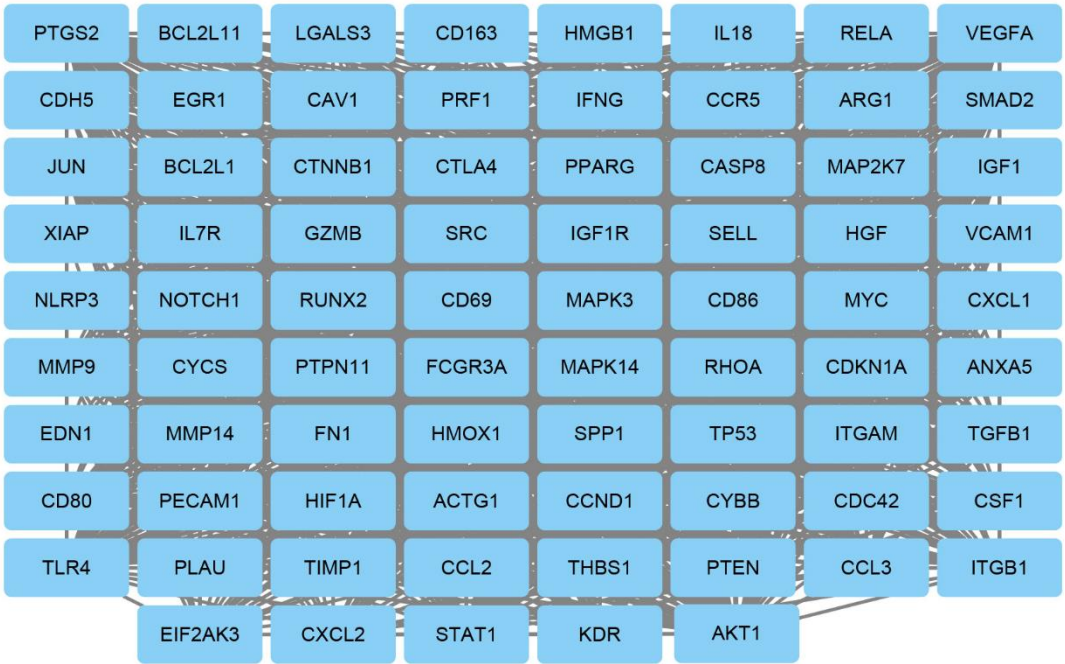

Supplementary Figure 1. PPIs network of potential key genes.

Supplement: Supplementary Figure 1 [file aging-16-205797-s001.pdf]
